# Specific Growth Rate Determines the Sensitivity of *Escherichia coli* to Lactic Acid Stress: Implications for Predictive Microbiology

**DOI:** 10.1155/2014/471317

**Published:** 2014-07-08

**Authors:** Roland Lindqvist, Gunilla Barmark

**Affiliations:** ^1^Division of Risk and Benefit Assessment, National Food Agency, 75126 Uppsala, Sweden; ^2^Department of Biomedical Sciences and Veterinary Public Health, Swedish University of Agricultural Sciences, 750 07 Uppsala, Sweden; ^3^Department of Microbiology, Swedish University of Agricultural Sciences, 750 07 Uppsala, Sweden; ^4^Department of Forest Products, Swedish University of Agricultural Sciences, 750 07 Uppsala, Sweden

## Abstract

This study tested the hypothesis that sensitivity of* Escherichia coli* to lactic acid at concentrations relevant for fermented sausages (pH 4.6, 150 mM lactic acid, *a*_*w*_ = 0.92, temperature = 20 or 27°C) increases with increasing growth rate. For* E. coli* strain 683 cultured in TSB in chemostat or batch, subsequent inactivation rates when exposed to lactic acid stress increased with increasing growth rate at harvest. A linear relationship between growth rate at harvest and inactivation rate was found to describe both batch and chemostat cultures. The maximum difference in T_90_, the estimated times for a one-log reduction, was 10 hours between bacteria harvested during the first 3 hours of batch culture, that is, at different growth rates. A 10-hour difference in T_90_ would correspond to measuring inactivation at 33°C or 45°C instead of 37°C based on relationships between temperature and inactivation. At similar harvest growth rates, inactivation rates were lower for bacteria cultured at 37°C than at 15–20°C. As demonstrated for* E. coli* 683, culture conditions leading to variable growth rates may contribute to variable lactic acid inactivation rates. Findings emphasize the use and reporting of standardised culture conditions and can have implications for the interpretation of data when developing inactivation models.

## 1. Introduction

Shiga-toxin-producing* Escherichia coli* (STEC) O157:H7 and several other STEC serotypes have been linked to serious illness outbreaks via food, for example, beef, minced beef, alfalfa sprouts, and fresh produce [1]. Acid foods, including fermented meats, mayonnaise, and unpasteurized juices, containing organic acids such as lactic and acetic acid have also been associated with outbreaks. This has been attributed to the acid stress response mechanisms of STEC O157:H7 strains leading to enhanced survival in these foods and in the gastrointestinal tract compared to other pathogens, for example [2]. However, a growing amount of evidence indicates that the extent of variation in acid survival among STEC strains is not different from the variation found within generic* E. coli* strains (e.g., [3, 4]). Indeed,* E. coli* has been described as an amateur acidophilic species [5].

*E. coli* survival during acid stress has been shown to be dependent on many factors, for example, growth phase, growth medium and pH, acid challenge medium and pH, temperature, acidulants, and preadaptation of strains [6–12]. In general, stationary phase batch cultures are more resistant to stress than exponential phase cultures. For* E. coli* O157:H7 cultured in different foods, survival during a subsequent acid challenge (pH = 3.0) was better for stationary phase cells than for exponential phase cells, except when cells were cultured in tomato and ginger, probably because of the lower pH values, 4.5-4.6, of these foods [13]. At growth temperatures below 20°C the stationary phase-specific higher survival was not observed.

The stationary phase-specific survival is often attributed to increases in intracellular levels of the *σ* ^*s*^ (RpoS) subunit of RNA polymerase which redirects mRNA synthesis making stationary phase cells more stress resistant [14]. Interestingly, specific growth rate, which decreases during the transition from exponential to stationary growth phase, was demonstrated to control the expression of RpoS and the general stress response in* E. coli* [15]. Thus, specific growth rate has the potential to control important aspects of* E. coli* physiology [16]. Experimentation using continuous culture of microorganisms (chemostat) enables studies under conditions of set of specific growth rates (equal to dilution rate, *D*, under steady state conditions) and defined and constant physicochemical conditions [17]. Using chemostat culturing it has been shown that increases in specific growth rates increase the sensitivity of* E. coli* to physical stresses such as thermal stress, UVA, and solar disinfection [18]. The dependence of lactic acid stress resistance on the physiological state of* E. coli* cells has to our knowledge been addressed only in terms of growth phase, that is, exponential and stationary phases, not in terms of growth rate.

The purposes of the present study were (i) to test the hypothesis that sensitivity of* E. coli* 683 to lactic acid stress at concentrations relevant for fermented sausages increases with increasing specific growth rate and (ii) to discuss the potential implications for predictive microbiology. Specifically, the effects of growth rate, medium composition, temperature, and growth phase on subsequent inactivation rates under lactic acid stress (150 mM, pH = 4.6, *a*_*w*_ = 0.92) were estimated for* E. coli* 683 cultured at a range of specific growth rates in continuous culture (chemostat). Experiments were also done in batch mode to investigate effects of harvest growth rates in different stages of the growth curve from inoculation to stationary phase on subsequent inactivation rates.

## 2. Materials and Methods

### 2.1. Bacterial Strain and Culture Conditions

A generic* Escherichia coli* strain (683) with good survival and growth capacity under conditions typical for fermented sausages was used [4, 19]. This strain is reported to have intermediate times to growth and similar inactivation rates when compared to those of three verocytotoxin producing* E. coli* outbreak strains (O103, O111, and O157) at lactic acid stress conditions [4]. The strain was stored at −80°C in TSB (Tryptone Soya Broth, Oxoid) with 20% glycerol. Prior to experiments the strain was streaked on TSA (Tryptone Soya Agar, Oxoid) and incubated overnight at 37°C. A single colony was picked from the plate and inoculated into TSB with 1% (filter-sterilized, Sigma-Aldrich) glucose (TSB + G) and cultured overnight at 37°C. The preculture was subcultured overnight before each experiment started. (i) Chemostat cultivation: a miniscale chemostat system as described in [20] was used in the continuous culture experiments with some modifications. The system consisted of peristaltic pumps, a water bath, and a glass vessel with a screw cap equipped with a butyl rubber septum. The septum was pierced by four needles, one for substrate supply, one needle for water saturated air supply, one for withdrawal of culture broth, and one for air efflux. The modifications included the use of a 25 mL (fast growth rates) or 50 mL (slow growth rates) glass flask (Schott Duran, Germany) with pierced screw caps and working volumes up to 14 or 40 mL, respectively, instead of a 17 mL Hungate tube. Also, the aeration rate used corresponded to approximately 5 instead of 2 volumes per volume per minute. In our set-up this was the flow rate above which the increase in final OD_600_ levelled off indicating that oxygen was not limiting for growth. Bacteria precultured as described above were diluted to an OD_600_ of 0.01 in the growth medium. The culture was grown for 5 hours in batch mode at 15, 20, or 37°C before continuous operation was initiated by feeding fresh TSB medium (100 or 25%) at the desired flow rate for 5 volume changes. During continuous culturing the effluent volume of medium and time were monitored and OD_600_ was measured at regular intervals. In a chemostat, growth rate is equal to the dilution rate at steady state, that is, at constant biomass or OD_600_, and the specific growth rate was estimated by dividing *F*, the flow rate (mL h^−1^), with *V*, the volume of medium (mL), in the chemostat, *μ* = *F*/*V* (h^−1^). (ii) Batch cultivation: a sufficient volume of the TSB + G preculture to obtain an optical density at 600 nm (OD_600_) of 0.01 or 0.1, corresponding to approximately 10^6^–10^8^ cfu/mL, was inoculated into 100% TSB or 25% TSB. The culture was incubated at 37°C with shaking at 160 rpm and samples for plate counts, optical density determination, and inactivation were taken at appropriate time intervals. In batch culture, the specific growth rate, *μ*, at the different times of sampling was estimated from the OD_600_ growth curve using five consecutive OD_600_ measurements, two before, two after, and the one at the sampling time, as described in [18]:

(1) $$\begin{array}{c} \mu =\frac{\Delta {\text{lnOD}}_{600}}{\Delta t},\;\text{where}\;t\;\text{is}\;\text{time}. \end{array}$$

### 2.2. Inactivation under Lactic Acid Stress

A sufficient volume to obtain an OD_600_ = 0.5 after centrifugation, resuspension, and dilution was harvested from chemostat or batch cultures. This volume was centrifuged at 3000 ×g for 15 minutes, and the pellet was dissolved in 5 mL peptone water (0.1% peptone, 8.5% NaCl). This solution was diluted 1 : 39 in a total volume of 20 mL of inactivation media to obtain an* E. coli* concentration of 10^6^-10^7^ CFU per mL. Inactivation medium consisted of BHI broth (Brain Heart Infusion, Oxoid) supplemented with 10.3% NaCl and 150 mM lactic acid (DL-lactic acid, 90%, VWR, Sweden) filter-sterilized (Filtropur S 0.2 *μ*m filter, Sarstedt, Germany) and adjusted to a pH of 4.6 with 1 M NaOH (Merck, Darmstadt, Germany). Survival at 20°C or 27°C was measured in triplicate eflasks. These inactivation temperatures are in the range commonly used for fermentation of cold-smoked sausages in Sweden [19]. At different time intervals the number of bacteria was quantified by plating decimal dilutions of the inactivation medium on TSA plates (Oxoid) and in some experiments also on violet red bile glucose agar (VRBGA, Oxoid) plates and incubating at 37°C. Colony forming units were counted after 24 hours.

### 2.3. Statistical Analyses and Inactivation Modelling

The initial part of inactivation curves, defined by data points within approximately the first day of inactivation, was used to estimate the inactivation rate by fitting the log linear model in the GInaFit software [21] to data.

Statistical tests were run using MINITAB statistical software version 15 (Coventry, UK). A significance level of 0.05 was used. General linear model (GLM) was used to analyse effects of growth rate, growth medium, temperature, culture method (batch or chemostat), inactivation temperature, and biomass (OD_600_) on inactivation rates. Multiple pairwise comparisons using the method of Tukey's test for differences of means were used to indicate significant effects of chemostat growth temperatures on subsequent inactivation rates. Student's *t*-test was used to test the effect of growth medium on OD_600_ (cell density).

## 3. Results

**Figure 1:**
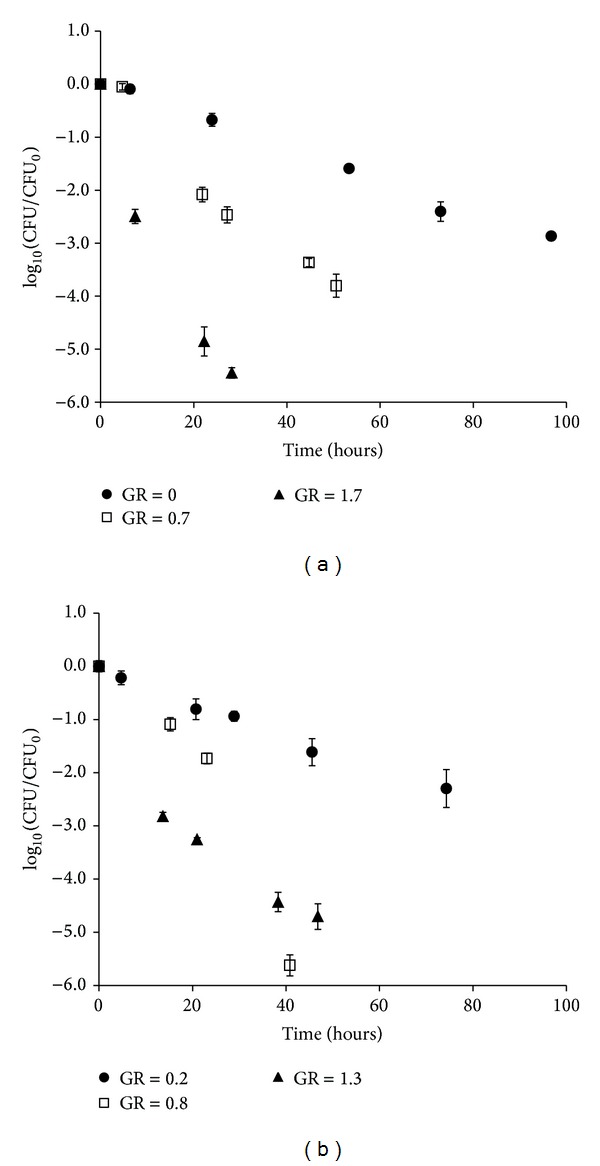
Sensitivity to lactic acid stress (150 mM HLac, pH = 4.6, 10.3% NaCl) at 27°C of* E. coli* 683 harvested at three different specific growth rates from (a) batch or (b) chemostat cultures at 37°C in TSB. Sensitivity was determined as CFU/CFU at time zero. GR = growth rate at harvest. Symbols indicate the mean of three replicates and error bars at the 95% confidence interval.

Most inactivation curves for bacteria harvested from chemostat and batch culture were log linear or log linear with a tail (Figure 1). The initial, linear part of the inactivation curves, defined by data points within the first day of inactivation, was used to estimate the inactivation rate by fitting the log linear model in GInaFit to data. Data from only the first 24 hours was fitted since it can be expected that the influence of preinactivation conditions is greatest during the initial phases of inactivation.

During lactic acid stress, estimated inactivation rates of bacteria cultured in chemostat were significantly greater when evaluated with selective VRBGA plates than with nonselective TSA plates (*P* < 0.05, *t*-test, paired). Inactivation rates evaluated at 27°C were significantly greater than at 20°C with both types of agar plates. In subsequent experiments, inactivation rates were investigated at 27°C and in some cases at 20°C (Table 1), and survival was quantified on TSA plates.

Cell biomass (OD_600_) in chemostat cultures at harvest varied between 0.5 and 1.7 (Table 1) but was not significantly affected by growth rate (*P* > 0.05, GLM) indicating that growth was not limited by oxygen at the higher growth rates. Mean OD_600_ in cultures grown in full strength TSB was 1.1 and in cultures grown in 25% TSB was 0.8 (*P* = 0.05, *t*-test). No significant effects of growth medium strength on subsequent survival during lactic acid stress (*P* > 0.05, GLM) were observed for cells cultured in chemostat or batch. Accordingly, results from experiments using 25% or 100% TSB as growth medium at 37°C were pooled in the following analysis to evaluate effects of growth rates at harvest on subsequent inactivation rates.

**Figure 2:**
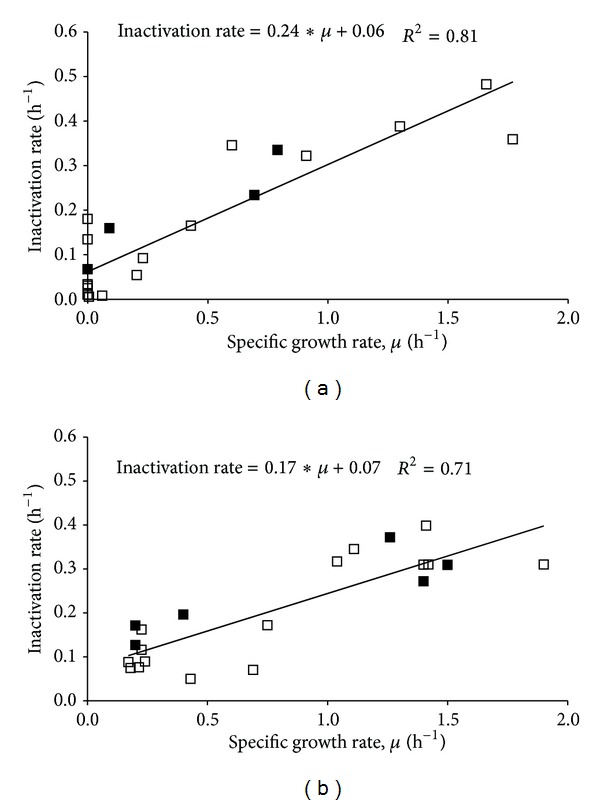
Relationship between estimated inactivation rate in BHI (27°C, pH = 4.6, 150 mM lactic acid, *a*_*w*_ = 0.92) and growth rate at harvest for* E. coli* 683 cultured at 37°C in 25% (□) or 100% (■) TSB: (a) batch culture and (b) chemostat culture. Lines and equations represent the best fit by linear regression.

*E. coli* were harvested from TSB batch cultures at different specific growth rates, that is, at different time points on the growth curve, and were exposed to lactic acid stress at 27°C (Table 1). There was a significant effect of growth rate on the subsequent inactivation rate (*P* < 0.001, GLM), and inactivation was slower for cells with lower specific growth rates than for faster growing cells (Figure 2(a)). The same pattern was observed for* E. coli* cells cultured in chemostat at similar growth rates, that is, dilution rates, and exposed to the same stress (Figure 2(b)).

There was a positive correlation between growth rates at harvest and subsequent inactivation rates estimated for* E. coli* cells cultured in batch (*r* = 0.90, Pearson) and chemostat (*r* = 0.85, Pearson), and data could be described by linear relationships (Figure 2). Interestingly, there was no significant effect of culture method (*P* = 0.18, GLM), and the same linear regression equation could be used to describe the relationship between growth rate and inactivation rate for both chemostat- and batch-grown cells:

(2) Inactivation  rate(h−1) =0.20∗Growth  rate(h−1)+0.07.

**Figure 3:**
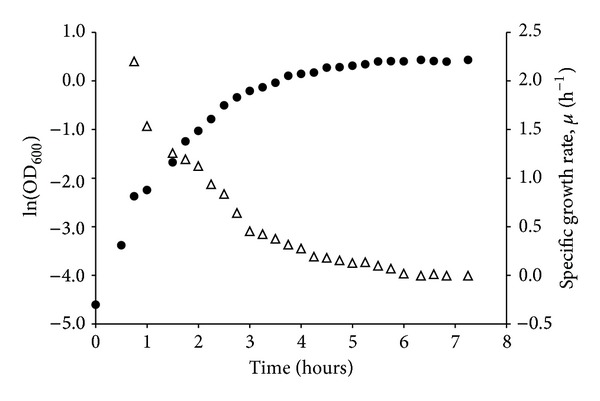
Growth curve of* E. coli* 683 in TSB batch culture (shaken Erlenmeyer flask) at 37°C (●, ln OD_600_). The specific growth rate *μ* (Δ, h^−1^) was calculated as the slope of five adjacent points.

Replicate inactivation rates determined for bacteria harvested at approximately the same growth rates varied less than 0.09 h^−1^ (chemostat) and 0.12 h^−1^ (batch) or within a factor of about two (Table 2). In comparison, the maximum difference in inactivation rates for bacteria harvested at all different growth rates was 0.35 h^−1^ (chemostat) and 0.47 h^−1^ (batch), respectively (Table 2). In relative terms, mean inactivation rates varied by a factor of 8 (0.40/0.05) and 48 (0.48/0.01) times, for* E. coli* cells cultured in chemostat and batch, respectively. Growth rates at harvest varied between 0.17 and 1.90 h^−1^ (chemostat) and between 0 and 1.77 h^−1^ (batch), respectively (Table 2). Thus, variation in growth rates at harvest may influence subsequent estimates of inactivation rates unless care is taken to control this parameter. Growth rates of* E. coli* cultured in batch in TSB medium estimated based on OD_600_ measurements decreased continuously until the stationary phase and the maximum difference during the initial 3 hours was ca 1.7 h^−1^ (Figure 3). This indicates the magnitude of variation in growth rates under the present conditions.

**Figure 4:**
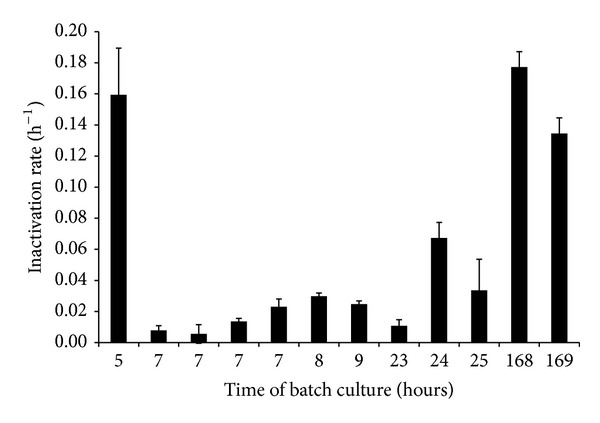
Estimated inactivation rates and standard error of the estimate (error bar) of “stationary phase”* E. coli* during lactic acid stress (150 mM HLac, pH = 4.6, *a*_*w*_ = 0.92) at 27°C. Data represents results from inactivation experiments with bacteria harvested after varying times of batch culturing in TSB at 37°C.

Inactivation rates of batch cultivated* E. coli* cells harvested in the “stationary phase” displayed a variation greater than that within a factor of two experimental variations (Figure 4) observed for replicate experiments at similar growth rates greater than zero (previous paragraph and Table 2). Further, when estimated inactivation rates of bacteria are compared based on when bacteria were harvested, expressed as the time from the start of the experiment, it is indicated that larger inactivation rates are associated with bacteria sampled early or late in the growth curve (Figure 4). These results suggest that cells sampled after 5 hours were not yet in stationary phase and that postgrowth processes become increasingly important for the sensitivity of* E. coli* to lactic acid stress during extended no-growth conditions.

Growth temperature at harvest also had a significant effect on* E. coli* survival during subsequent lactic acid stress (*P* < 0.05, GLM). Mean inactivation rates of bacteria cultured at similar growth rates (0.17–0.25 h^−1^) in chemostat at 15 and 20°C were significantly greater (*P* < 0.05, Tukey's test) than for bacteria cultured at 37°C.

## 4. Discussion

The interplay between* E. coli* cells and the environment is reflected in the physiology and growth rate of the bacterial population. The expression of more than 300 genes, many implicated in virulence and stress tolerance, has been found to be modulated in a growth dependent manner in* E. coli* O157 [22]. The hypothesis that growth rate of* E. coli* 683 at harvest has an impact on subsequent sensitivity to lactic acid stress in terms of inactivation rates was tested at conditions relevant for the production of fermented sausages. Using both chemostat and batch cultured bacteria it was shown that* E. coli* 683 sensitivity to lactic acid increased with increasing growth rate at harvest.

The increase of inactivation rates with increasing growth rates at harvest was not totally consistent and occasionally inactivation rates of bacteria were lower than those observed at lower harvest growth rates (Table 1, Figure 2). It is possible that a limiting maximum inactivation rate exists and that this is approached at around 0.4 h^−1^ at the higher harvest growth rates (Figure 2, Table 1). Using fully equipped bioreactors, Berney et al. [18] showed, in agreement with the present study, that sensitivity of* E. coli* to stress, that is, thermal, UVA, and solar disinfection, also increased with growth rate. Slow-growing bacteria (*μ* = 0.08 h^−1^) that were exposed to mild heat treatment at 48°C had T_90_ (time to a one-log reduction) values of 2.6 hours, whereas T_90_ for faster growing cells (*μ* = 0.9 h^−1^) was 0.2 hours [18]. In that study, effects of harvest growth rates on the shapes of inactivation curves were observed but such effects were not investigated or observed in the present study.

Although uncertainty remains around the estimated linear relationships between growth and inactivation rates for batch or chemostat data results indicate that variations in growth rates explain a large part, here up to 80%, of the variation in observed inactivation rates (Figure 2). In Figure 3, maximum and minimum growth rates during the first 3 hours of batch growth are 2.2 and 0.45 h^−1^. Using (2), this translates into inactivation rates of 0.51 and 0.16 h^−1^, respectively. This corresponds to T_90_ (the time for a one-log reduction) of 4.5 and 14.4 hours, respectively. Thus, the maximum difference in estimated times for a one-log reduction using bacteria in “late exponential phase” is around 10 hours under these conditions. Present results also illustrate the variation in growth rates that may exist due to use of batch cultured bacteria in early and late stationary phase (Figure 4). Based on Figure 4 it may be speculated that the first estimated inactivation rates reflect the transition of batch cultured bacteria from exponential to stationary phase.

Thus, growth rates at harvest have an effect on subsequent* E. coli* 683 inactivation rates under lactic acid stress, in agreement with results for other E. coli strains exposed to thermal stress, UVA, and solar disinfection [18]. To put the effect of harvest growth rate on* E. coli* inactivation during lactic acid stress in perspective, a comparison can be made with the effect of temperature on inactivation rates under similar growth-preventing conditions. A quantitative relationship between temperature and* E. coli* inactivation rates under growth-preventing conditions has been reported [23]. The relationship reflects conditions in fermented meats and analogous broth-based systems. For* E. coli* at 37°C, a T_90_ of 17 hours can be calculated based on the relationship. A 10-hour difference in T_90_, as calculated for “late exponential phase bacteria” in our example above, would correspond to measuring inactivation at 33°C (longer T_90_) or 45°C (shorter T_90_) instead of 37°C.

As illustrated and discussed in Berney et al. [18] and the present study, it is possible that some of the variation observed in inactivation studies may be due to and interpreted as being a consequence of nonstandardised culture conditions leading to variation in bacterial growth rates. This emphasizes the importance of using and reporting standardised culture conditions when developing data and models for predictive microbiology.

The increase in inactivation rates with inactivation temperature reported for* E. coli* [24] and other bacteria [19, 23] under no-growth conditions mimicking fermented meats was observed also in the present study. Inoculum (harvest) growth temperature effects on subsequent inactivation rates are less clear. A greater survival, in terms of the times for a one-log reduction in peptone with lactic acid, was reported for two out of three* E. coli* O157:H7 strains cultured at 10°C compared to 37°C [25]. The results in that study and in Cebrián et al. [26] suggest that the growth temperature history effect on* E. coli* survival may depend on both the strain and stress factor evaluated. Data in those studies was generated using batch cultures and thus the possible influence of temperature on bacterial growth rates and growth stages was not evaluated. In contrast, in the present study using* E. coli* cultured in chemostat at similar growth rates, it was observed that subsequent inactivation rates decreased with increasing growth temperature. The latter suggests that when effects of temperature on growth rates are controlled by chemostat culture, subsequent survival is less at lower than at higher harvest temperatures. However, no definite conclusion about temperature history effects on inactivation can be drawn from this comparison but the issue is important since it may have implications for food manufacturing processes [27].

Growth medium in the present study, that is, different strength TSB, did not have a significant impact on subsequent inactivation rates. In comparison, McQuestin et al. [11] reported that the inactivation suspension medium affected* E. coli* survival during nonthermal stress based on a comparison between minimal medium and different complex nutrient broths. The direction of this effect varied depending on the stress factor investigated [11]. In our study, the effect of inactivation suspension medium on the survival of bacteria was not evaluated.

## 5. Conclusions

Using chemostat- and batch-grown cultures it was shown that under the present conditions* E. coli* 683 sensitivity to lactic acid increased with increasing harvest growth rates. The same linear relationship between growth rate at harvest and subsequent inactivation rate under lactic acid stress could be used to describe both chemostat and batch growth cultures of* E. coli* 683. Under the present conditions and at similar growth rates, subsequent inactivation rates decreased with harvest growth temperatures. Thus, when effects of growth rate and temperature are controlled during chemostat culture, subsequent survival after low harvest temperature (15–20°C) is less than at high harvest temperature (37°C). The results for* E. coli* 683 and lactic acid suggest, in agreement with results for other* E. coli* strains and physical stress factors [18], that the interpretation of inactivation experiments may be different whether effects of growth rates are considered or not, since observed effects may be due to culture conditions leading to variable growth rates. The findings emphasize the use and reporting of standardised culture conditions when developing data and models for predictive microbiology and can have implications for the interpretation of data used for predictive microbiology.

## Figures and Tables

**Table 1:** Summary of estimated inactivation rates during lactic acid stress (pH 4.6, 150 mM lactic acid, *a*_*w*_ = 0.92) at 20 and 27°C for strain *E. coli* 683 following culture in TSB medium in chemostat or batch. Surviving cells were quantified on TSA plates.

Exp. Number^1^	Growth					Inactivation					
	Medium^2^	Method	Temp. (°C)	Biomass (OD_600_)	Rate (h^−1^)	Rate at 27°C (h^−1^)	SE^4^ of estimated rate	RMSE^4^ of fit	Mean rate at 20°C (h^−1^)	SE of estimated rate	RMSE of fit
32	TSB 25%	Chemostat	37	1.29	0.17	0.09	0.01	0.121	0.03	0.005	0.053
33	TSB 25%	Chemostat	37	1.26	0.18	0.08	0.01	0.142	0.01	0.003	0.060
27	TSB 25%	Chemostat	15	0.60	0.20	No result	No result	No result	0.34	0.04	0.549
28	TSB 25%	Chemostat	15	0.98	0.20	0.92	0.12	0.525	0.61	0.05	0.230
12	TSB	Chemostat	37	1.56	0.20	0.13	0.01	0.127	0.03	0.01	0.070
16	TSB	Chemostat	37	0.99	0.20	0.17	0.01	0.066	0.06	0.005	0.063
26	TSB 25%	Chemostat	20	1.62	0.22	0.42	0.06	0.832	0.18	0.02	0.348
34	TSB 25%	Chemostat	37	0.52	0.22	0.08	0.003	0.050	ND	ND	ND
21	TSB 25%	Chemostat	15	0.65	0.23	0.43	0.04	0.138	0.34	0.03	0.097
24_1	TSB 25%	Chemostat	37	0.67	0.23	0.12	0.01	0.074	ND	ND	ND
24_2	TSB 25%	Chemostat	37	0.67	0.23	0.16	0.02	0.242	ND	ND	ND
17	TSB 25%	Chemostat	37	0.80	0.24	0.09	0.01	0.118	0.04	0.004	0.050
20	TSB 25%	Chemostat	20	1.19	0.25	0.32	0.03	0.368	0.21	0.004	0.044
13	TSB	Chemostat	37	0.92	0.40	0.20	0.02	0.059	0.02	0.003	0.087
36	TSB 25%	Chemostat	37	1.00	0.43	0.05	0.01	0.099	ND	ND	ND
38	TSB 25%	Chemostat	37	0.57	0.69	0.07	0.02	0.321	ND	ND	ND
39	TSB 25%	Chemostat	37	0.73	0.75	0.17	0.01	0.082	ND	ND	ND
41	TSB 25%	Chemostat	20	0.82	0.65	0.25	0.03	0.259	ND	ND	ND
18	TSB 25%	Chemostat	37	0.88	1.04	0.32	0.03	0.334	0.17	0.01	0.153
22	TSB 25%	Chemostat	37	1.00	1.11	0.34	0.03	0.296	0.16	0.02	0.196
15	TSB	Chemostat	37	0.75	1.26	0.37	0.03	0.373	0.17	0.01	0.141
14	TSB	Chemostat	37	1.75	1.40	0.27	0.01	0.155	0.14	0.01	0.099
31	TSB 25%	Chemostat	37	0.56	1.40	0.31	0.02	0.234	0.16	0.01	0.091
23	TSB 25%	Chemostat	37	0.69	1.41	0.40	0.01	0.046	0.14	0.01	0.164
37	TSB 25%	Chemostat	37	0.65	1.42	0.31	0.03	0.331	ND	ND	ND
11	TSB	Chemostat	37	0.64	1.50	0.31	0.01	0.107	ND	ND	ND
35	TSB 25%	Chemostat	37	0.55	1.90	0.31	0.01	0.130	ND	ND	ND

10_1	TSB	Batch	37	0.21	0.79	0.33	0.02	0.202	ND	ND	ND
10_2	TSB	Batch	37	1.61	0.69	0.23	0.02	0.223	ND	ND	ND
10_3	TSB	Batch	37	2.39	0.09	0.16	0.03	0.362	ND	ND	ND
10_4	TSB	Batch	37	2.8	0^3^	0.07	0.01	0.085	ND	ND	ND
19_1	TSB 25%	Batch	37	0.08	1.30	0.39	0.08	0.912	ND	ND	ND
19_2	TSB 25%	Batch	37	1.30	0.20	0.05	0.005	0.054	ND	ND	ND
19_3	TSB 25%	Batch	37	1.44	0	0.01	0.01	0.066	ND	ND	ND
19_4	TSB 25%	Batch	37	1.43	0	0.03	0.02	0.065	ND	ND	ND
29_1	TSB 25%	Batch	37	0.09	1.77	0.36	0.04	0.531	ND	ND	ND
29_2	TSB 25%	Batch	37	0.98	0.23	0.09	0.01	0.074	ND	ND	ND
29_3	TSB 25%	Batch	37	1.26	0	0.01	0.002	0.032	ND	ND	ND
29_4	TSB 25%	Batch	37	0.91	0	0.01	0.004	0.052	ND	ND	ND
29_5	TSB 25%	Batch	37	1.60	0	0.18	0.01	0.054	ND	ND	ND
30_1	TSB 25%	Batch	37	0.11	1.66	0.48	0.04	0.486	ND	ND	ND
30_2	TSB 25%	Batch	37	0.96	0.43	0.17	0.01	0.152	ND	ND	ND
30_3	TSB 25%	Batch	37	1.40	0	0.02	0.005	0.114	ND	ND	ND
30_4	TSB 25%	Batch	37	1.37	0	0.03	0.002	0.159	ND	ND	ND
30_5	TSB 25%	Batch	37	1.33	0	0.02	0.002	0.129	ND	ND	ND
40_1	TSB 25%	Batch	37	0.04	0.91	0.32	0.04	0.434	ND	ND	ND
40_2	TSB 25%	Batch	37	0.76	0.60	0.35	0.01	0.067	ND	ND	ND
40_3	TSB 25%	Batch	37	1.51	0.06	0.01	0.003	0.024	ND	ND	ND
40_4	TSB 25%	Batch	37	0.96	0	0.13	0.01	0.059	ND	ND	ND

ND: not determined.

^1^Chemostat results are sorted by growth rate and batch results by experiment and sampling time after start of culturing, that is, along the growth curve.

^2^100% or 25% TSB.

^3^Zero growth rate indicates stationary phase in batch experiments.

^4^SE: standard error; RMSE: root mean squared error (the square root of the mean of the sum of squared errors).

**Table 2:** Inactivation rates during lactic acid stress (150 mM HLac, pH = 4.6, *a*_*w*_ = 0.92) at 27°C of *E. coli* 683 harvested at different growth rates. Cells were grown in TSB at 37°C in chemostat or batch culture.

Method	Growth rate at harvest (h^−1^)	Number of experiments	Inactivation rate (h^−1^)			
			Mean (SD)	Minimum	Maximum	CV
Chemostat						
Replicate^1^ experiments	1.4	3	0.34 (0.05)	0.31	0.40	0.15
	0.2	8	0.11 (0.04)	0.08	0.17	0.36
All experiments	0.17–1.90	21	0.21 (0.12)	0.05	0.40	0.57
Batch						
Replicate experiments	1.7	2	0.42 (0.09)	0.36	0.48	0.21
	0.7	2	0.28 (0.07)	0.23	0.33	0.25
	0.2	2	0.07 (0.03)	0.05	0.09	0.43
All experiments	0–1.77	21	0.16 (0.16)	0.01	0.48	1.00

^1^Experiments at similar growth rates.

SD: standard deviation.

CV: coefficient of variation.
